# Increased risk of secondary malignancies after autologous transplantation in R/R follicular lymphoma: Long‐term results from FIL‐FLAZ12 Phase III trial

**DOI:** 10.1002/hem3.70453

**Published:** 2026-08-11

**Authors:** Rita Tavarozzi, Andrea Evangelista, Manuela Zanni, Chiara Consoli, Alessandra Tucci, Barbara Botto, Silvia Bolis, Pietro Lauzzana, Vittorio Ruggero Zilioli, Benedetta Puccini, Annalisa Arcari, Vincenzo Pavone, Gloria Margiotta‐Casaluci, Monica Tani, Federica Cavallo, Antonio Pinto, Chiara Ghiggi, Domenico Pastore, Paolo Corradini, Andrés José María Ferreri, Angelo Palmas, Caterina Patti, Francesca Re, Fabio Benedetti, Giuseppe Milone, Stefano Luminari, Elsa Pennese, Elisa Bossi, Carola Boccomini, Antonella Anastasia, Chiara Bottelli, Donato Mannina, Samantha Pozzi, Catello Califano, Luca Arcaini, Flavio Falcinelli, Giuseppe Pietrantuono, Ilaria Del Giudice, Sara Usai, Jacopo Olivieri, Filippo Gherlinzoni, Attilio Olivieri, Tommasina Perrone, Fabrizio Pomponi, Giovannino Ciccone, Umberto Vitolo, Marco Ladetto

**Affiliations:** ^1^ Department of Translational Medicine University of Eastern Piedmont Novara Italy; ^2^ SCDU of Hematology AOU SS Antonio e Biagio e Cesare Arrigo Alessandria Italy; ^3^ Unit of Clinical Epidemiology AOU Città Della Salute E Della Scienza Di Torino and CPO Piemonte Turin Italy; ^4^ Department of Molecular Biotechnology and Health Sciences, Hematology Division University of Torino Turin Italy; ^5^ Department of Hematology Spedali Civili Brescia Italy; ^6^ Struttura Complessa Ematologia AOU Città della salute e della scienza di Torino Turin Italy; ^7^ Division of Haematology SC Ematologia Fondazione Irccs San Gerardo dei Tintori Monza Italy; ^8^ Division of Hematology, Clinica Ematologica, Centro Trapianti e Terapie Cellulari Carlo Melzi DISM, Azienda Ospedaliero Universitaria S. M. Misericordia Udine Italy; ^9^ Division of Haematology ASST Grande Ospedale Metropolitano Niguarda Milan Italy; ^10^ Department of Haematology University of Florence Florence Italy; ^11^ Division of Hematology AUSL of Piacenza Piacenza Italy; ^12^ Hematology Unit Ospedale Guglielmo da Saliceto Piacenza Italy; ^13^ SC of Hematology A. O. C. Panico‐U. O. C Ematologia e Trapianto Tricase Italy; ^14^ Division of Hematology AOU Maggiore della Carità di Novara Novara Piedmont Italy; ^15^ Hematology Unit, Department of Oncology and Hematology “Santa Maria delle Croci” Hospital Ravenna Italy; ^16^ Department of Hematology Istituto Nazionale Tumori Istituto di Ricovero e Cura a Carattere Scientifico “Fondazione G Pascale” Naples Italy; ^17^ IRCCS Azienda Ospedaliera Metropolitana Divisione di Ematologia e Terapie Cellulari Genova Italy; ^18^ Division of Hematology Ospedale Antonio Perrino Brindisi Italy; ^19^ Division of Hematology IRCCS Istituto Nazionale Tumori Milan Italy; ^20^ Lymphoma Unit IUniversity Vita‐Salute San Raffaele Milan Italy; ^21^ Strategic Program on Lymphomas IRCCS San Raffaele Scientific Institute Milan Italy; ^22^ Unità di Ematologia e Trapianto di Midollo Osseo, San Francesco Hospital Nuoro Italy; ^23^ SCDU of Hematology “Villa Sofia – Cervello” Hospital Palermo Italy; ^24^ Department of Hematology A. O. U. di Parma Parma Italy; ^25^ Department of Medicine, Section of Hematology and Bone Marrow Transplant Unit University of Verona Verona Italy; ^26^ Division of Hematology and Program for Hematopoietic Transplantation Azienda Ospedaliera Policlinico Vittorio Emanuele Catania Italy; ^27^ Hematology Azienda Unità Sanitaria Locale IRCCS of Reggio Emilia Reggio Emilia Italy; ^28^ SCDU of Hematology Presidio Ospedaliero Pescara Pescara Italy; ^29^ Hematology Unit Papardo Hospital Messina Messina Italy; ^30^ Dipartimento di Scienze Mediche e Chirurgiche Materno‐Infantili e dell'Adulto Università di Modena e Reggio Emilia Modena Italy; ^31^ Hematology Hospital “Andrea Tortora” Pagani Italy; ^32^ Department of Molecular Medicine University of Pavia Pavia Italy; ^33^ Division of Hematology Fondazione IRCCS Policlinico San Matteo Pavia Italy; ^34^ Hematology and Bone Marrow Transplantation Azienda Ospedaliera Di Perugia Perugia Italy; ^35^ IRCCS CROB Referral Cancer Center of Basilicata Rionero in Vulture Italy; ^36^ Hematology, Department of Translational and Precision Medicine Sapienza University of Rome Rome Italy; ^37^ Unit of Hematology and Bone Marrow Transplant AO Brotzu ‐ Ospedale Oncologico Businco Cagliari Italy; ^38^ Clinica Ematologica Azienda Sanitaria Universitaria Friuli Centrale Udine Italy; ^39^ Division of Hematology Ca' Foncello Hospital Treviso Italy; ^40^ SCDU of Hematology Ospedale Regionale delle Marche Ancona Italy; ^41^ Department of Emergency and Organ Transplantation D. E. T. O., Hematology Section University of Bari Bari Italy; ^42^ Division of Hematology U.O.C. di Ematologia ‐ AULSS8 Berica Vicenza Italy; ^43^ Hematology IRCCS Candiolo Cancer Institute Turin Italy

In the rituximab era, autologous stem cell transplantation (ASCT) has historically been employed as consolidation therapy in relapsed/refractory (R/R) follicular lymphoma (FL), largely based on retrospective series and registry studies.^1^, ^2^, ^3^, ^4^ Despite growing concerns regarding long‐term toxicity,^5^, ^6^ contemporary data from the European Society for Blood and Marrow Transplantation (EBMT) registry indicate that ASCT continues to be used in a non‐negligible fraction of FL patients,^7^, ^8^, ^9^ in the absence of randomized comparisons with less intensive approaches. The FIL‐FLAZ12 trial (NCT01827605) was designed to address this gap by directly comparing ASCT with an anti‐CD20 radioimmunotherapy (RIT) as consolidation in this setting.^10^ Initial analyses showed no advantage for ASCT in progression‐free (PFS) or overall survival (OS), while revealing greater acute toxicity.^10^ We provide here the updated results of this trial, after a median follow‐up (mFU) of 103 months, offering a comprehensive evaluation of long‐term survival, causes of deaths and the occurrence of secondary malignancies (SMs).

Eligible patients were 18–65 years old with histologically confirmed FL, R/R after at least one prior line of systemic therapy, without major comorbidities or history of active prior malignancy.^10^

After three courses of investigator‐selected immunochemotherapy (R‐CHOP, R‐DHAP, R‐FM, R‐ICE, R‐IEV, or R‐bendamustine), responders were randomized 1:1 to ASCT (BEAM/FEAM conditioning) or RIT (rituximab plus 90Y‐ibritumomab tiuxetan). Both arms received eight courses of rituximab maintenance after response evaluation. The primary endpoint was PFS. Full study details and primary analysis have been previously published.^10^

During this long‐term analysis, data collection focused on PFS, OS, cause of death, and incidence of SMs, which were systematically updated across participating centers. Cumulative incidence of SMs and cause‐specific mortality were analyzed using competing‐risks methods (Gray's test and Fine–Gray models), with death and causes of death, respectively, treated as competing events.^11^

Between August 2012 and September 2019, 164 patients were enrolled across Fondazione Italiana Linfomi (FIL) centers in Italy. Of these, 141 responders were randomized: 70 to ASCT and 71 to RIT. After randomization, 61 in the ASCT arm and 63 in the RIT arm proceeded beyond mobilization and constituted the per‐protocol population.^10^ The median age was 57 years (49–62), 55% of patients were male, 57% Stage IV, and 20% presented bulky disease. Baseline characteristics have been detailed previously.^10^

With a mFU of 103 months, the 84‐month PFS and OS from consent in the overall population were 44% (95% CI: 36–52; Figure S1A) and 77% (95% CI: 69–83; Figure S1B), respectively. In the randomized cohort, there was no significant difference between the two consolidation strategies. Patients receiving ASCT had an 84‐month PFS from randomization of 46% (95% CI: 33–57) and 49% (95% CI: 37–60) in the RIT arm (hazard ratio [HR] = 0.98, 95% CI: 0.62–1.55; P = 0.92—Figure 1A). Similarly, OS from randomization outcomes overlapped: 84‐month was 78% (95% CI: 65.30–86.30) for ASCT versus 77% (95% CI: 65.00–85.60) for RIT (HR = 0.92, 95% CI: 0.47–1.80; P = 0.80—Figure 1B). Multivariable analyses identified male sex (HR = 2.35, 95% CI: 1.48–3.73; P < 0.001), elevated LDH (HR = 1.71, 95% CI: 1.03–2.84; P = 0.038), extranodal disease (HR = 1.79, 95% CI: 1.09–2.92; P = 0.021), and POD24 (HR = 2.13, 95% CI: 1.33–3.43; P = 0.002) as independent predictors of inferior PFS (Table S1). Exploratory subgroup analyses suggested a potential ASCT benefit in the small cohort of patients with primary refractory disease (*n* = 16; P = 0.002), while limited‐stage patients appeared to have a better outcome with RIT (P = 0.006) (Figure S2). Notably, POD24 patients did not benefit from ASCT, further questioning its value in a high‐risk setting (Figure S2).

**Figure 1:**
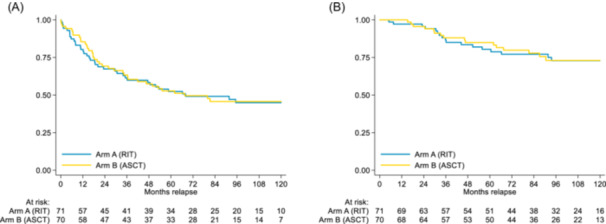
**(A) Progression‐free survival (PFS) from randomization and (B) overall survival (OS) from randomization.** ASCT, autologous stem cell transplantation; RIT, radioimmunotherapy.

At 84 months, lymphoma‐specific mortality was 15.2% (95% CI: 7.90–22.50) versus 12.0% (95% CI: 5.40–18.60) (HR = 1.04, 95% CI: 0.44–2.48; P = 0.928—Figure S3A) and non‐lymphoma‐specific mortality was 6.8% (95% CI: 1.70–11.90) versus 10.7% (95% CI: 4.40–17.00) (HR = 0.76, 95% CI: 0.27–2.19; P = 0.615—Figure S3B) in the ASCT and RIT arms, respectively. A detailed summary of causes of death is provided in Table S2.

During the follow‐up period, 20 SMs (12%) were reported in the whole population, 19 SMs (14%) in the intention‐to‐treat (ITT) cohort and 16 (12%) in the per‐protocol population (Figure S4).

The 5‐year (60‐month) cumulative incidence from consent was 8.6% (95% CI: 4.8–13.8%) in the enrolled population (Figure S5). In the primary ITT analysis, SM incidence from randomization was higher after ASCT than after RIT (5‐year cumulative incidence 13.5% [95% CI: 5.20–21.80] vs. 6.0% [95% CI: 0.20–11.80]; Fine–Gray HR = 4.18, 95% CI: 1.38–12.70; P = 0.012—Figure 2A), corresponding to an absolute risk difference of 7.5%, consistent after adjustment for induction regimen (HR = 4.55, 95% CI: 1.52–13.63; P = 0.007). When stratified by type of malignancy, the risk after ASCT was driven predominantly by solid tumors: the 5‐year cumulative incidence from randomization was 7.6% [95% CI: 1.10–14.10] in the ASCT arm compared with 1.5% [95% CI: 0.00–4.50] in the RIT arm (absolute risk difference, 6.1%; HR = 10.89, 95% CI: 1.36–87.21; Gray's test P = 0.004—Figure S5A). The incidence of hematologic SMs from randomization showed no evidence of a difference between the two groups (5.9% [95% CI: 0.20–11.50] in the ASCT group vs. 4.5% [95% CI: 0.00–9.50] in the RIT group; absolute risk difference, 1.4%; HR = 1.77, 95% CI: 0.43–7.34; Gray's test P = 0.427—Figure S5B). These findings were confirmed in the per‐protocol sensitivity analysis (5‐year cumulative incidence 11.8% [95% CI: 5.20–21.40] in ASCT group vs. 6.8% in RIT arm [95% CI: 2.20–15.10]; Fine–Gray HR = 3.26, 95% CI: 1.04–10.21—P = 0.043; Figure 2B), again driven by solid tumors (5‐year cumulative incidence 6.9% [95% CI: 0.30–13.50] vs. 1.7% [95% CI: 0.00–5.10]; HR = 9.78, 95% CI: 1.19–80.08; Gray's test P = 0.008—Figure 2C), while there was no evidence of a difference in the incidence of hematologic SMs between the two arms (4.9% [95% CI: 0.00–10.40] vs. 5.1% [95% CI: 0.00–10.70]; HR = 1.04, 95% CI: 0.21–5.15; Gray's test P = 0.957—Figure 2D).

**Figure 2:**
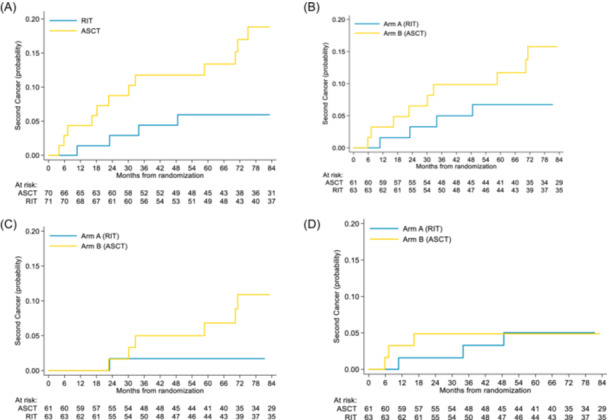
**Five‐year (60‐month) cumulative incidences of secondary malignancies (SMs) in different study population. (A)** Second cancer any‐type in intention‐to‐treat (ITT) population. **(B)** SMs of any‐type in the per‐protocol population (PP). **(C)** Solid SMs in the PP. **(D)** Hematological SMs in the PP. ASCT, autologous stem cell transplantation; RIT, radioimmunotherapy.

Of note, only a minority of SMs occurred after FL progression: 5/20 (25%), 4/19 (21%), and 2/16 (12.5%) in the enrolled, ITT, and per‐protocol populations, respectively. The complete list of SMs across all study populations, including type and timing, is reported in Table S3. Nine of the 20 SMs (45%) were fatal, 7 (44%) of which occurred in the per‐protocol population (Table S2).

Within the ASCT arm, FEAM was associated with a non‐significantly higher SM risk versus BEAM (HR = 2.14; 95% CI: 0.44–10.31; P = 0.345). These findings remained consistent after adjustment for induction treatment regimen (Table S4). A time‐dependent Fine–Gray model showed no significant change in the ASCT effect across follow‐up periods (P = 0.689) (Table S5). Hematologic SMs appeared earlier than solid tumors, with a median latency of 22 months from randomization, compared with 59 months for solid tumors, highlighting distinct temporal patterns in their development.

This long‐term analysis demonstrates that ASCT provides no benefit compared with RIT in PFS or OS for patients with R/R FL responding to salvage immunochemotherapy. The lack of a plateau in the PFS curve underscores its minimal, if any, curative potential. At the time of trial design, POD24 had not yet been established as a prognostic criterion.^12^ Even if a heterogeneous retrospective analysis by Jurinovic et al. suggested a benefit from ASCT in POD24 patients,^13^ our prospective data did not confirm this finding compared to an ASCT‐free consolidation platform.

Importantly, the exceptionally long mFU of 103 months enabled the detection of late‐occurring solid tumors (median latency 59 months) that would have been missed at shorter timepoints; to our knowledge, this is the first randomized study reporting a signal of increased SMs risk following ASCT versus RIT, consistent with retrospective studies reporting 5‐year cumulative incidences of 6%–7% and 10‐year incidences up to 17.5% after ASCT in FL.^8^, ^9^ The observed excess suggests a substantial genotoxic burden of high‐dose chemotherapy, although the small sample size, heterogeneity of events, and exploratory nature of the analysis warrant cautious interpretation.

In the current landscape, characterized by highly active and less toxic options such as CAR T‐cell therapies^14^, ^15^, ^16^, ^17^ and bispecific antibodies,^18^, ^19^, ^20^ the continued use of ASCT should be carefully weighed against its long‐term safety profile. Novel therapies have yet to prove long‐term curative potential, but hold promise for superior efficacy and lower toxicity^20^ compared to ASCT, which has demonstrated clear limits in this setting.

## AUTHOR CONTRIBUTIONS

**Rita Tavarozzi**: Conceptualization; writing—original draft; writing—review and editing; formal analysis; data curation; supervision; methodology; validation; project administration. **Andrea Evangelista**: Conceptualization; writing—original draft; writing—review and editing; validation; methodology; formal analysis; supervision. **Manuela Zanni**: Writing—review and editing; writing—original draft; conceptualization; methodology. **Chiara Consoli**: Writing—review and editing. **Alessandra Tucci**: Writing—review and editing. **Barbara Botto**: Writing—review and editing. **Silvia Bolis**: Writing—review and editing. **Pietro Lauzzana**: Writing—review and editing. **Vittorio Ruggero Zilioli**: Writing—review and editing. **Benedetta Puccini**: Writing—review and editing. **Annalisa Arcari**: Writing—review and editing. **Vincenzo Pavone**: Writing—review and editing. **Gloria Margiotta‐Casaluci**: Writing—review and editing. **Monica Tani**: Writing—review and editing. **Federica Cavallo**: Writing—review and editing. **Antonio Pinto**: Writing—review and editing. **Chiara Ghiggi**: Writing—review and editing. **Domenico Pastore**: Writing—review and editing. **Paolo Corradini**: Writing—review and editing. **Andrés José María Ferreri**: Writing—review and editing. **Angelo Palmas**: Writing—review and editing. **Caterina Patti**: Writing—review and editing. **Francesca Re**: Writing—review and editing. **Fabio Benedetti**: Writing—review and editing. **Giuseppe Milone**: Writing—review and editing. **Stefano Luminari**: Writing—review and editing. **Elsa Pennese**: Writing—review and editing. **Elisa Bossi**: Writing—review and editing. **Carola Boccomini**: Writing—review and editing. **Antonella Anastasia**: Writing—review and editing. **Chiara Bottelli**: Writing—review and editing. **Donato Mannina**: Writing—review and editing. **Samantha Pozzi**: Writing—review and editing. **Catello Califano**: Writing—review and editing. **Luca Arcaini**: Writing—review and editing. **Flavio Falcinelli**: Writing—review and editing. **Giuseppe Pietrantuono**: Writing—review and editing. **Ilaria Del Giudice**: Writing—review and editing. **Sara Usai**: Writing—review and editing. **Jacopo Olivieri**: Writing—review and editing. **Filippo Gherlinzoni**: Writing—review and editing. **Attilio Olivieri**: Writing—review and editing. **Tommasina Perrone**: Writing—review and editing. **Fabrizio Pomponi**: Writing—review and editing. **Giovannino Ciccone**: Writing—review and editing. **Umberto Vitolo**: Writing—review and editing; writing—original draft; investigation; conceptualization; methodology; supervision; project administration. **Marco Ladetto**: Conceptualization; investigation; funding acquisition; writing—original draft; writing—review and editing; validation; methodology; formal analysis; project administration; supervision; data curation.

## CONFLICT OF INTEREST STATEMENT

R.T.—AbbVie, AstraZeneca, Novartis, BeOne, Roche, Sobi, and Lilly. V.R.Z.—AB: AbbVie, AstraZeneca, Kite/Gilead, Novartis, Roche, Sobi, and Takeda; speakers bureau: AbbVie, AstraZeneca, Incyte, Janssen, Lilly, Sobi, and Takeda; consultant: Roche; research support: Kite/Gilead; and travel expenses/others: AbbVie, Beigene, Janssen, Lilly, Roche, and Takeda. S.L.—AD Board o consulente per Roche, BeOne, Incyte, AbbVie, Novartis, Kite, BMS, and Regeneron. Speaker bureau per Roche, BMS, Kite, Regeneron, and Invyte. Travel grant da Roche and BeOne. LA—AstraZeneca, Novartis, Kite/Gilead, Beigene, and AbbVie (speaker bureau); Roche, Janssen‐Cilag, Incyte, EUSA Pharma, Celgene/Bristol Myers Squibb, Kite/Gilead, Novartis, and BMS (participation on a Data Safety Monitoring Board or Advisory Board); Roche and AstraZeneca (support for attending meetings and/or travel). M.L.—AbbVie, Amgen, ADC Therapeutics, Sobi, BeOne, BMS, Eusapharma, GSK, Gentili, Gilead/Kite, Novartis, Incyte, Janssen, Jazz, Lilly, Regeneron, Roche, Ellipses, MSD, Genmab, and AstraZeneca.

## ETHICS STATEMENT

The study was conducted in accordance with the principles of the Declaration of Helsinki and Good Clinical Practice guidelines and was approved by the local Ethics Committee. Written informed consent was obtained from all patients prior to enrollment.

## FUNDING

This work was supported by Fondazione Italiana Linfomi and by a competitive grant from AIFA (Cod.: FARM9RCP52). Further support derived from the grant no. MCL 7005‐24 of the Leukemia and Lymphoma Society (LLS), Progetto di Ricerca Sanitaria Finalizzata 2021 (RF‐2021‐12371972) and AIL AL‐ASTI ODV. Open access publishing facilitated by Università degli Studi del Piemonte Orientale Amedeo Avogadro, as part of the Wiley ‐ CRUI‐CARE agreement.

## Supporting information

Supporting Information.

## Data Availability

The data that support the findings of this study are available on request from the corresponding author. The data are not publicly available due to privacy or ethical restrictions.
