# Supplementary material for: Increased risk of secondary malignancies after autologous transplantation in R/R follicular lymphoma: Long‐term results from FIL‐FLAZ12 Phase III trial
Source: Hemasphere. 2026 Aug 11;10(8):e70453. doi: 10.1002/hem3.70453 (PMC13460027; doi:10.1002/hem3.70453)
Supplement: Supplementary file 1 [file HEM3-10-e70453-s001.pdf]

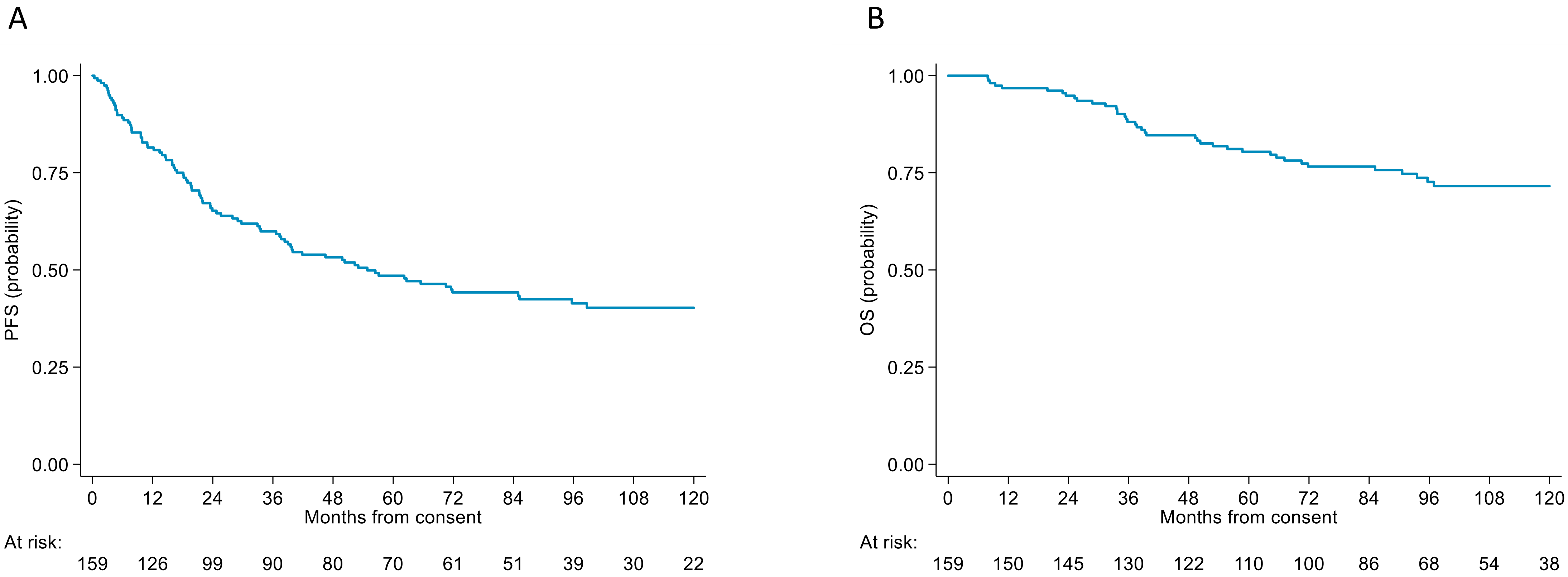

Figure S1 - A: Progression free survival from consent. B: Overall survival from consent

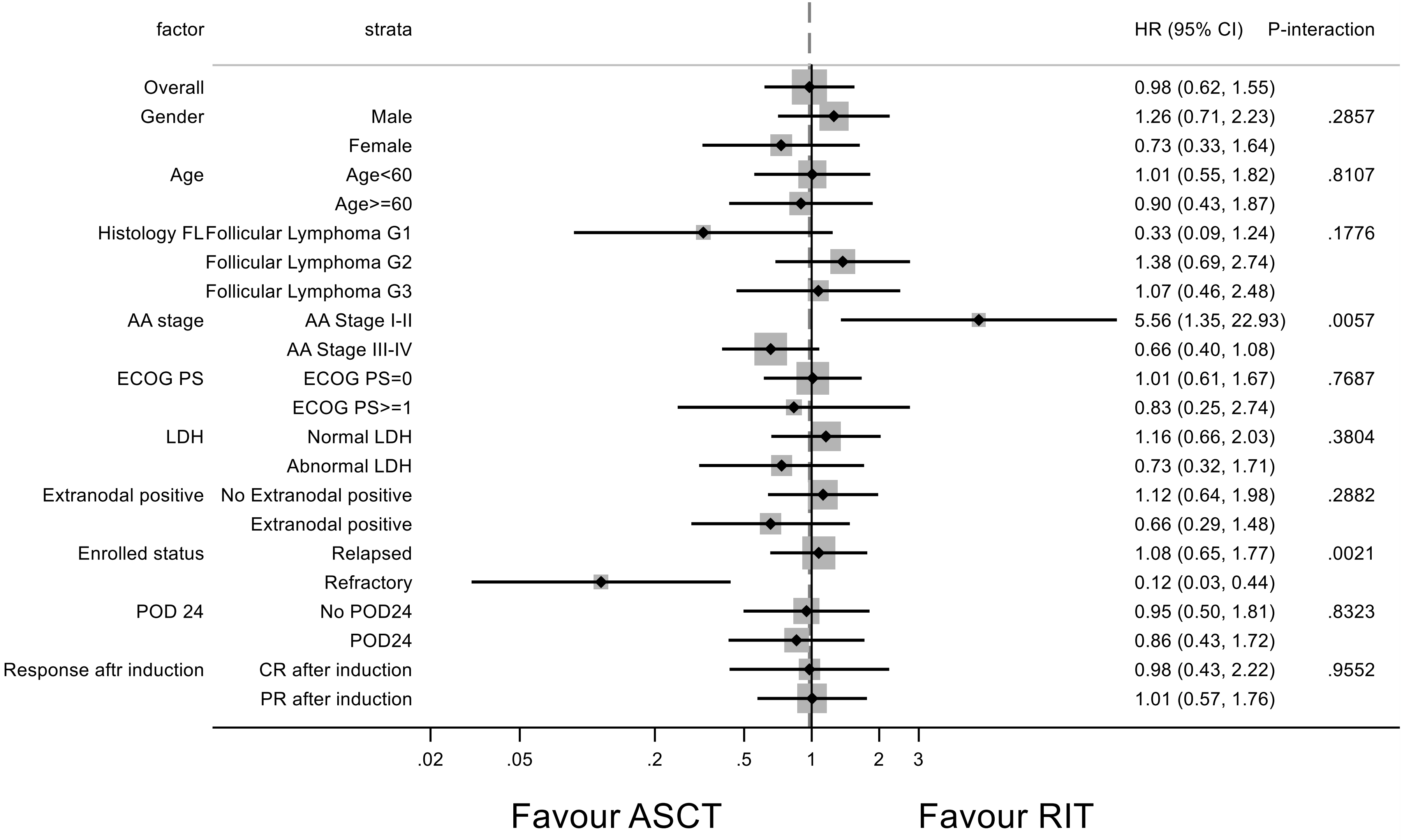

Figure S2 - Subgroup analysis of progression-free survival

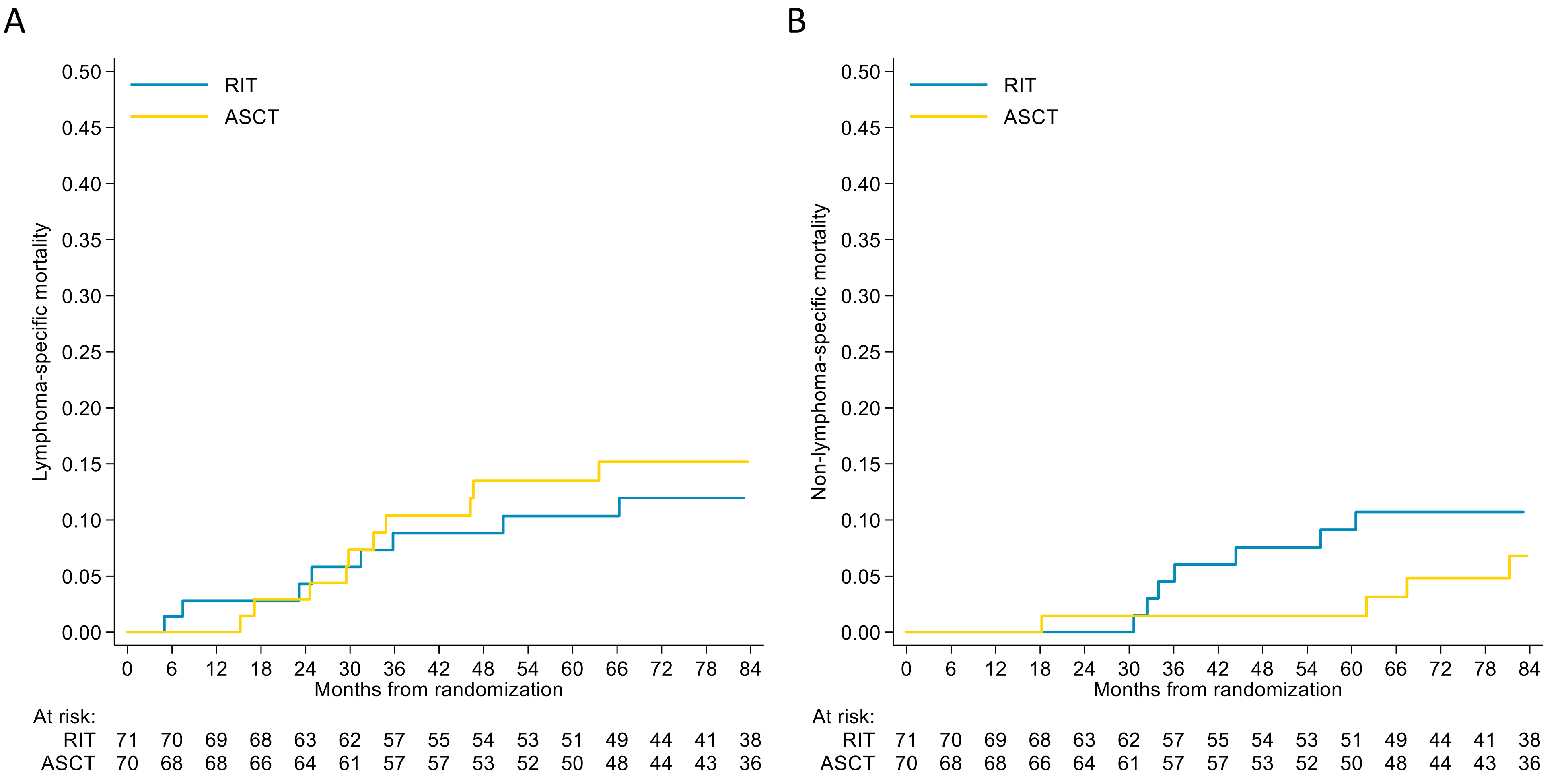

Figure S3 – A. Lymphoma-specific mortality up to 84 months from randomization. B. Non-lymphoma-specific mortality up to 84 months from randomization.

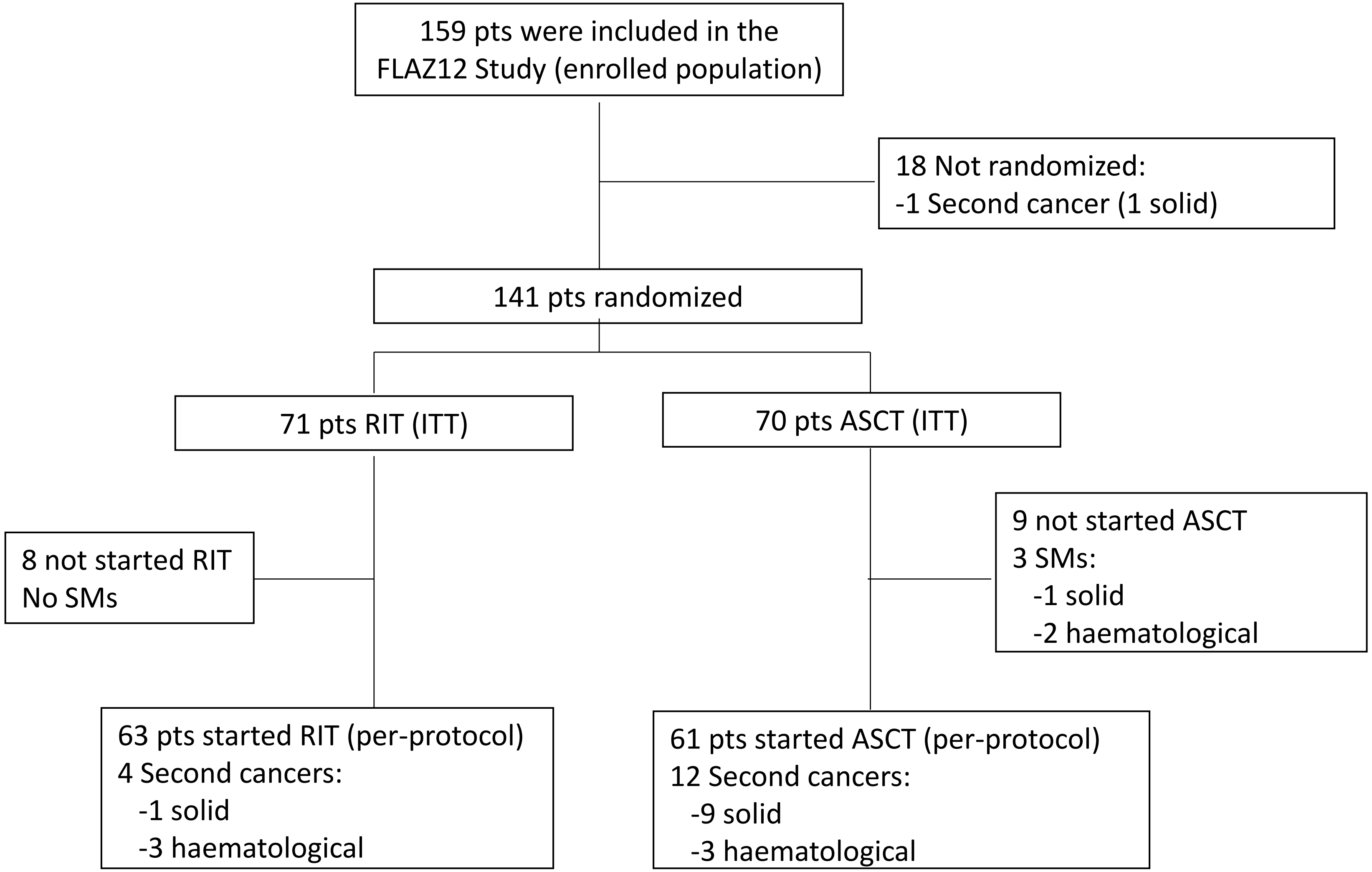

Figure S4 : Study flow chart and incidence of second malignancies. PTS: patients. SMs: secondary malignancies

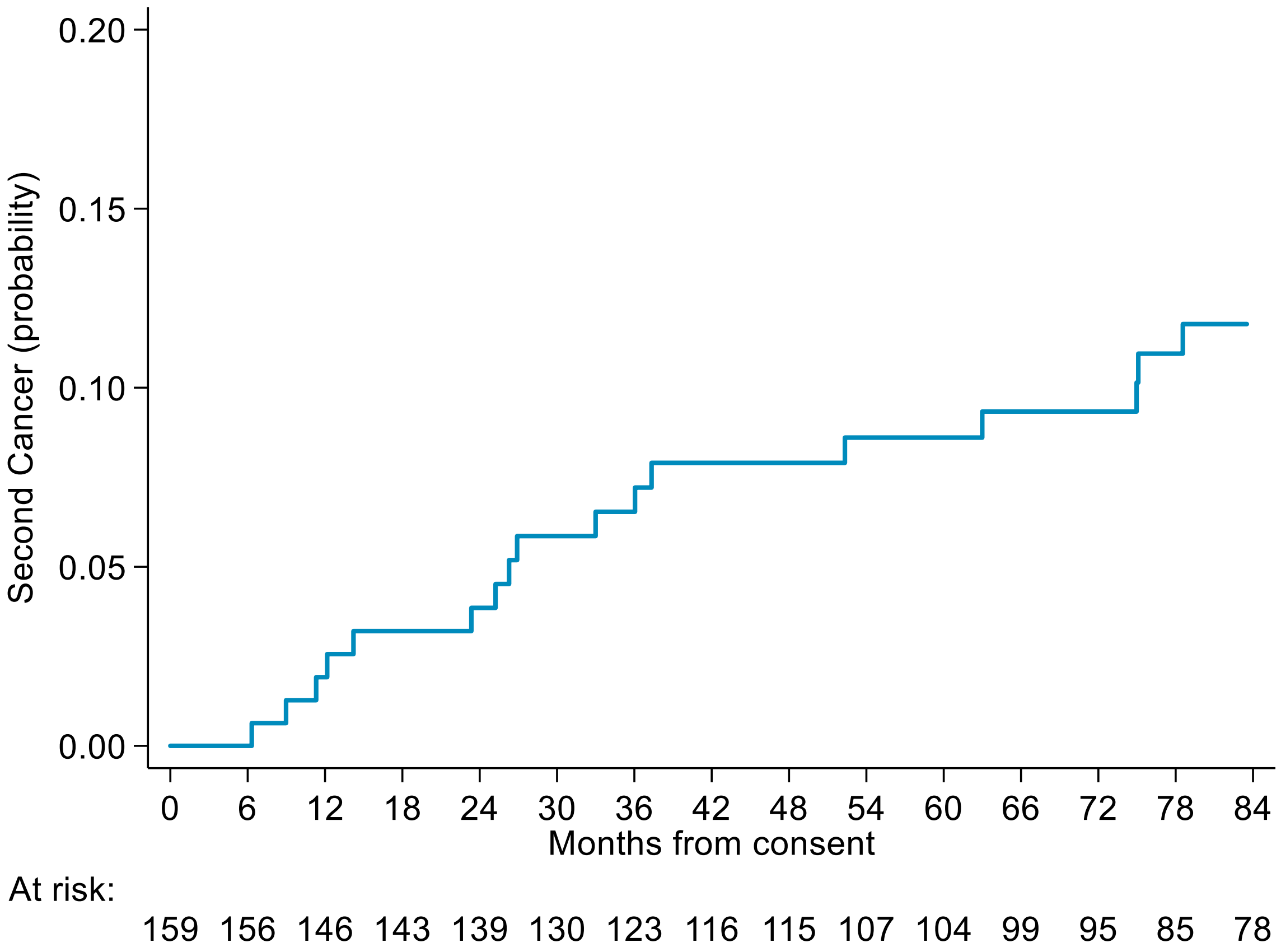

Figure S5- 5 years (60-months) cumulative incidence of SMs in the whole enrolled population (any type)

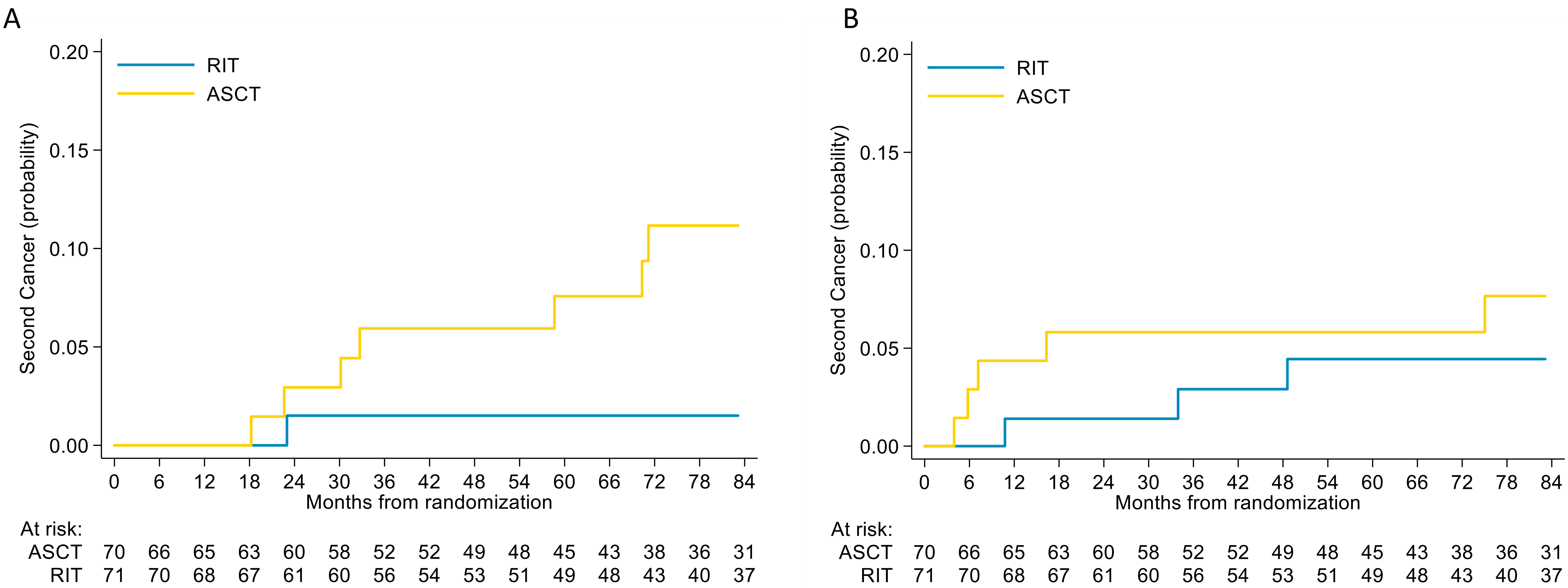

Figure S5 - Five-year (60-month) cumulative incidence of second malignancies (SMs) in the ITT population, by type. (A) Solid second malignancies. (B) Hematological second malignancies.

	HR	95% CI	p-value
Age at consent (years)	1.01	0.99–1.04	0.312
Male sex	2.35	1.48–3.73	<0.001
Ann Arbor stage III–IV	1.85	0.98–3.48	0.058
ECOG PS ≥1	0.99	0.56–1.76	0.977
High LDH	1.71	1.03–2.84	0.038
≥2 extranodal sites	1.79	1.09–2.92	0.021
Systemic symptoms	1.18	0.65–2.15	0.579
POD24 (Yes)	2.13	1.33–3.43	0.002

Table S1 - Multivariable Cox regression analysis of predictors of progression-free survival

N	Arm	Death cause
1	Non Randomized	Lymphoma
2	Non Randomized	Lymphoma
3	Non Randomized	Lymphoma
4	Non Randomized	Infection during induction
5	Non Randomized	Lymphoma
6	Non Randomized	Lymphoma
7	Arm A (RIT)	Lymphoma
8	Arm A (RIT)	Lymphoma
9	Arm A (RIT)	Lymphoma
10	Arm A (RIT)	Lymphoma
11	Arm A (RIT)	Lymphoma
12	Arm A (RIT)	Secondary neoplasm
13	Arm A (RIT)	Infection (Sars-CoV-2 infection complications)
14	Arm A (RIT)	Infection (Sars-CoV-2 infection complications)
15	Arm A (RIT)	Lymphoma
16	Arm A (RIT)	Lymphoma
17	Arm A (RIT)	Infection (Sars-CoV-2 infection complications)
18	Arm A (RIT)	Secondary neoplasm
19	Arm A (RIT)	Senectus
20	Arm A (RIT)	Secondary neoplasm
21	Arm A (RIT)	Other - Cause of death unknown (lost to follow-up)
22	Arm A (RIT)	Lymphoma
23	Arm A (RIT)	Lymphoma
24	Arm A (RIT)	Lymphoma
25	Arm B (ASCT)	Lymphoma
26	Arm B (ASCT)	Lymphoma
27	Arm B (ASCT)	Lymphoma
28	Arm B (ASCT)	Lymphoma
29	Arm B (ASCT)	Lymphoma
30	Arm B (ASCT)	Lymphoma
31	Arm B (ASCT)	Secondary neoplasm
32	Arm B (ASCT)	Secondary neoplasm
33	Arm B (ASCT)	Secondary neoplasm
34	Arm B (ASCT)	Secondary neoplasm
35	Arm B (ASCT)	Secondary neoplasm
36	Arm B (ASCT)	Lymphoma
37	Arm B (ASCT)	Lymphoma
38	Arm B (ASCT)	Secondary neoplasm
39	Arm B (ASCT)	Lymphoma
40	Arm B (ASCT)	Lymphoma

Table S2 – Cause of deaths

N	ARM	Cancer Type	Months from randomization
1	Not randomized population	Prostate	NA
2	ASCT	MDS	3.9
3	ASCT	MDS	5.8
4	ASCT	MDS	7.2
5	RIT	AML	10.8
6	ASCT	MDS	16.3
7	ASCT	Oral Carcinoma	18.2
8	ASCT	Bladder	22.6
9	RIT	Breast	23.0
10	ASCT	Bladder	30.2
11	ASCT	Bladder	32.7
12	RIT	AML	33.9
13	RIT	MDS	48.6
14	ASCT	HCC	58.7
15	ASCT	Local advanced cutaneous cancer	70.3
16	ASCT	Colon	71.2
17	ASCT	T-cell Lymphoma	75.0
18	ASCT	Adenocarcinoma	86.8
19	ASCT	Mandibola	88.1
20	ASCT	Breast	110.9

Abbreviations: MDS, myelodysplastic syndrome; AML, acute myeloid leukemia; HCC, hepatocellular carcinoma; ASCT, autologous stem cell transplantation; RIT, radioimmunotherapy; Bladder, urothelial bladder carcinoma; Colon, colorectal adenocarcinoma; Breast, breast carcinoma; Prostate, prostate adenocarcinoma; Mandibola, mandibular squamous cell carcinoma; Adenocarcinoma, pulmonary adenocarcinoma; Oral carcinoma, oral squamous cell carcinoma; Locally advanced cutaneous cancer, locally advanced cutaneous squamous cell carcinoma; T-cell lymphoma, peripheral T-cell lymphoma.

Table S3 - Time to onset of second malignancies (months from randomization), by treatment arm and cancer type

Competing risk: death without secondary malignancy. Reference arm: RIT.

Analysis	HR	95% CI	p-value
Overall secondary malignancies			
Unadjusted	4.18	1.38–12.7	0.012
Adjusted for induction regimen	4.55	1.52–13.63	0.007
Solid tumors			
Unadjusted	10.89	1.36–87.21	0.004
Adjusted for induction regimen	11.47	1.48-88.87	0.020
Hematologic malignancies			
Unadjusted	1.77	0.43–7.34	0.427
Adjusted for induction regimen	2.05	0-49-8.52	0.323

Table S4 — Fine–Gray model for secondary malignancy incidence: unadjusted and adjusted analyses (ITT population).

	HR	95% CI	p
Overall effect (entire follow-up)	4.18	1.38–12.68	0.012
Period-specific effects (time-dependent model)*			
0–12 months	2.93	0.40–21.54	0.29
12–24 months	3.82	1.19–12.27	0.024
>24 months	4.98	1.16–21.39	0.031

*Per-period HR increase = 1.30, 95% CI: 0.36–4.78; p = 0.689

Table S5. Time-dependent hazard ratios (HR) for ASCT vs RIT, second malignancy risk. Fine-Gray model
